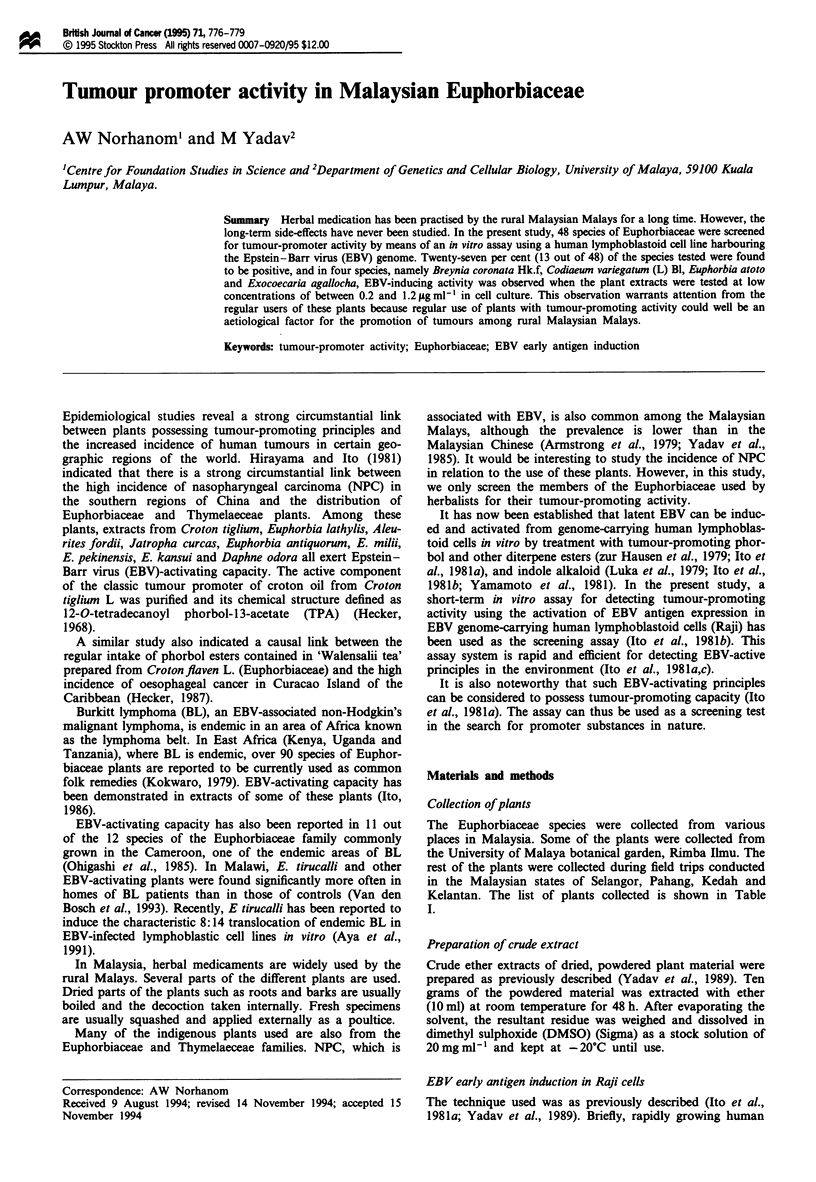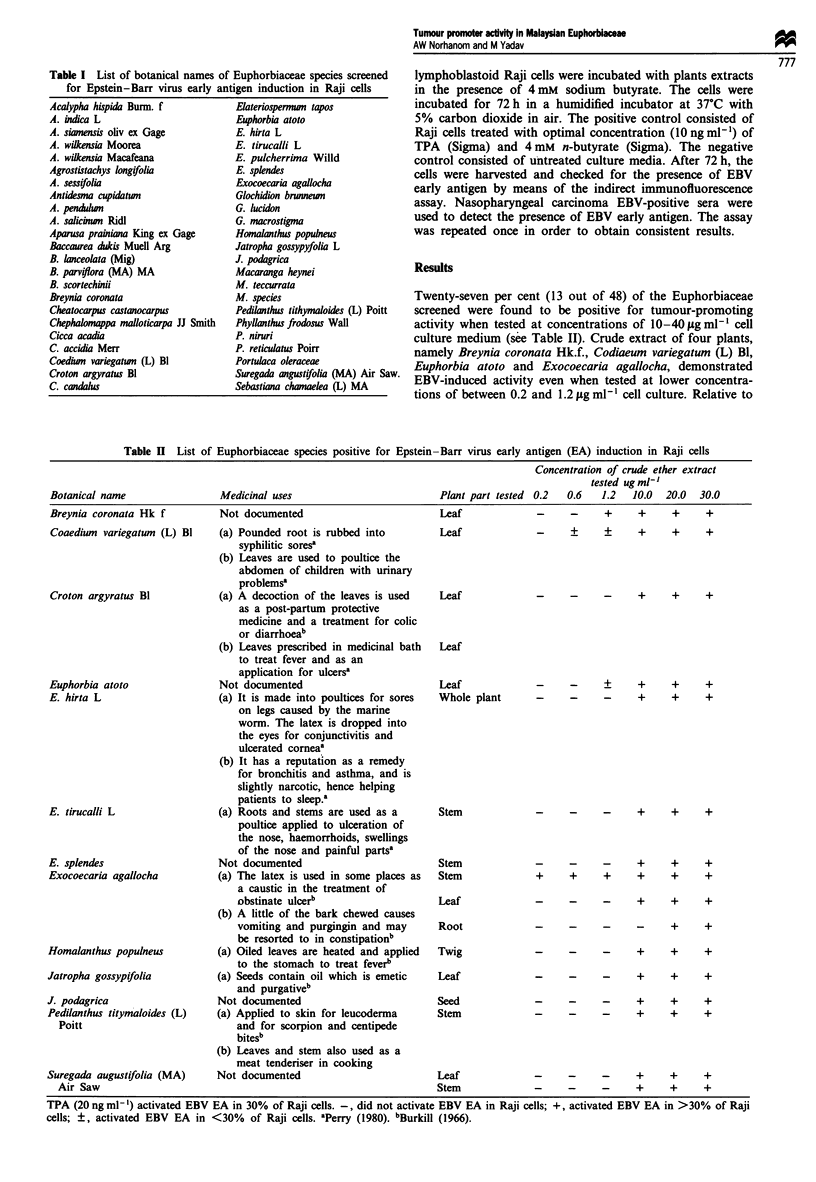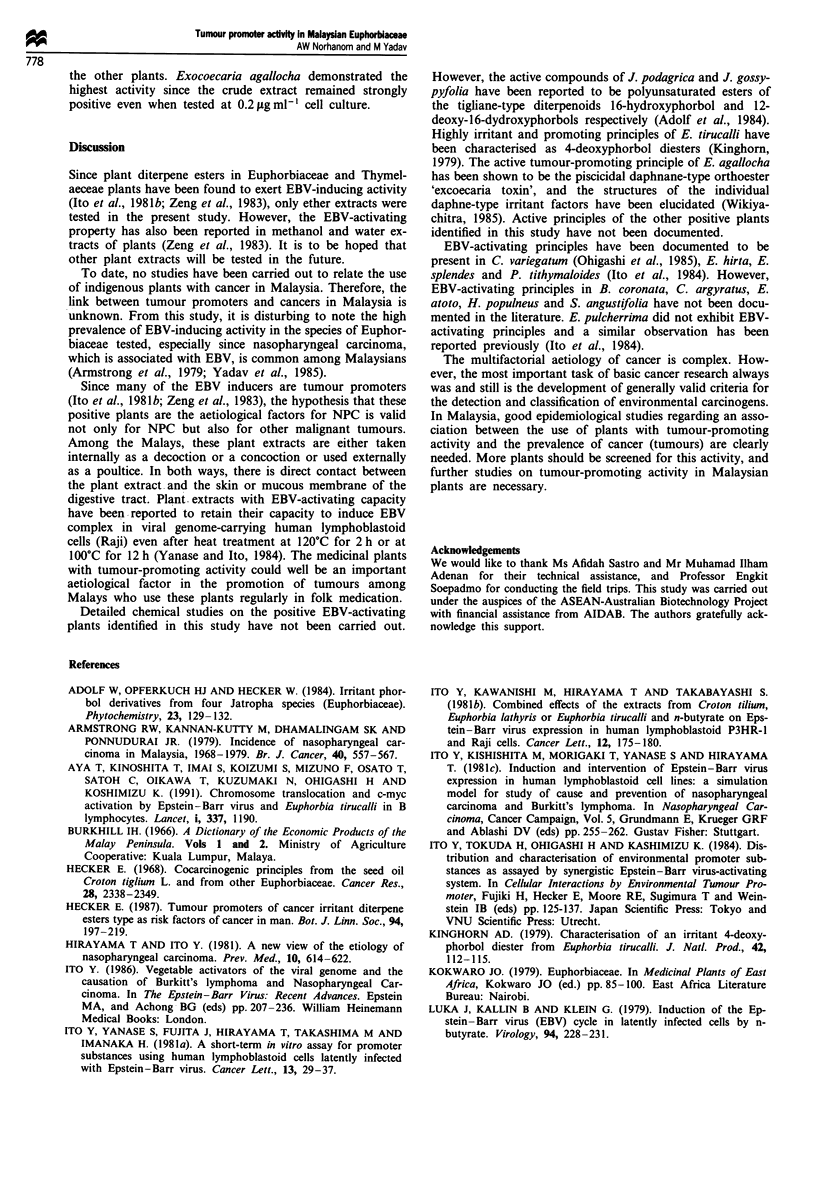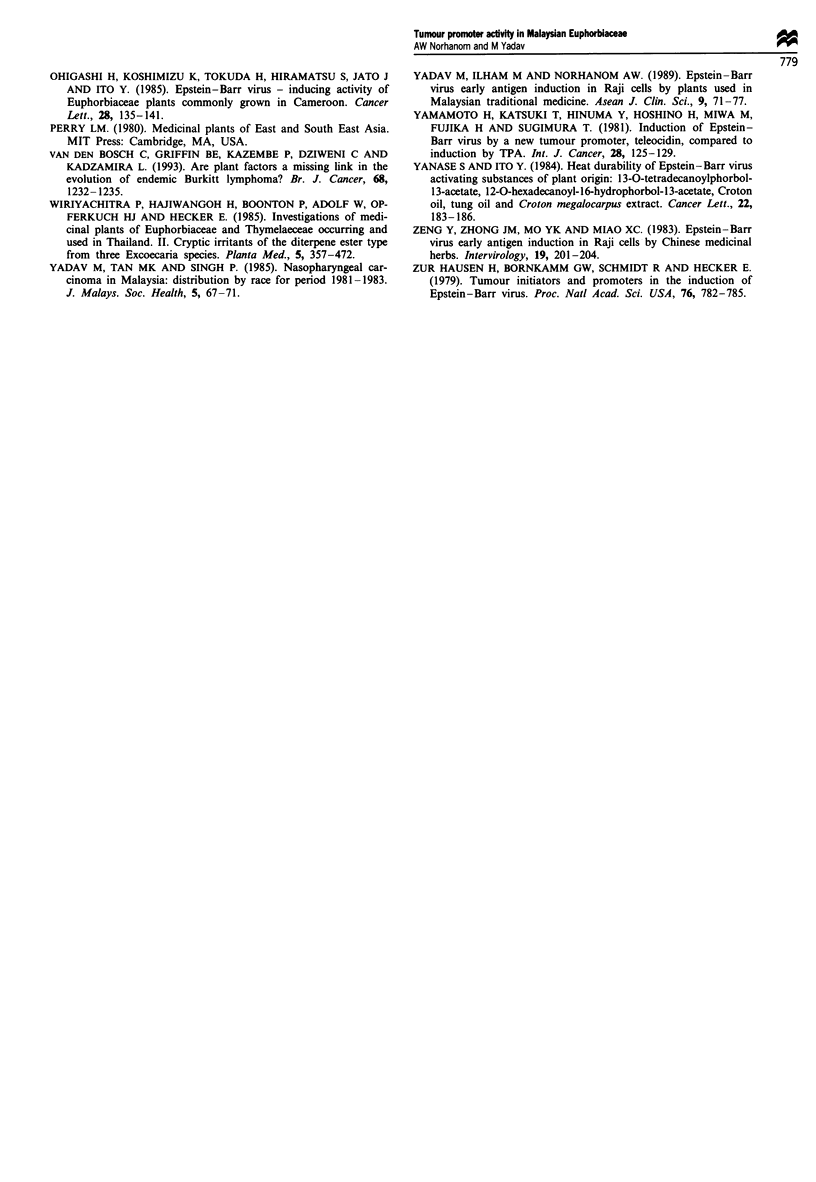# Tumour promoter activity in Malaysian Euphorbiaceae.

**DOI:** 10.1038/bjc.1995.150

**Published:** 1995-04

**Authors:** A. W. Norhanom, M. Yadav

**Affiliations:** Centre for Foundation Studies in Science, University of Malaya, Kuala Lumpur.

## Abstract

Herbal medication has been practised by the rural Malaysian Malays for a long time. However, the long-term side-effects have never been studied. In the present study, 48 species of Euphorbiaceae were screened for tumour-promoter activity by means of an in vitro assay using a human lymphoblastoid cell line harbouring the Epstein-Barr virus (EBV) genome. Twenty-seven per cent (13 out of 48) of the species tested were found to be positive, and in four species, namely Breynia coronata Hk.f, Codiaeum variegatum (L) Bl, Euphorbia atoto and Exocoecaria agallocha, EBV-inducing activity was observed when the plant extracts were tested at low concentrations of between 0.2 and 1.2 micrograms ml-1 in cell culture. This observation warrants attention from the regular users of these plants because regular use of plants with tumour-promoting activity could well be an aetiological factor for the promotion of tumours among rural Malaysian Malays.


British Journal of Cancer (1995) 71, 776-779
© 1995 Stockton Press All rights reserved 0007-0920/95 $12.00

### Tumour promoter activity in Malaysian Euphorbiaceae

AW Norhanom[1] and M Yadav[2]

[1]*Centre for Foundation Studies in Science and* [2]*Department of Genetics and Cellular Biology, University of Malaya, 59100 Kuala Lumpur, Malaya.*

**Summary** Herbal medication has been practised by the rural Malaysian Malays for a long time. However, the long-term side-effects have never been studied. In the present study, 48 species of Euphorbiaceae were screened for tumour-promoter activity by means of an *in vitro* assay using a human lymphoblastoid cell line harbouring the Epstein-Barr virus (EBV) genome. Twenty-seven per cent (13 out of 48) of the species tested were found to be positive, and in four species, namely *Breynia coronata* Hk.f, *Codiaeum variegatum* (L) Bl, *Euphorbia atoto* and *Exocoecaria agallocha*, EBV-inducing activity was observed when the plant extracts were tested at low concentrations of between 0.2 and 1.2 $\mu g\,ml^{-1}$ in cell culture. This observation warrants attention from the regular users of these plants because regular use of plants with tumour-promoting activity could well be an aetiological factor for the promotion of tumours among rural Malaysian Malays.

**Keywords:** tumour-promoter activity; Euphorbiaceae; EBV early antigen induction

Epidemiological studies reveal a strong circumstantial link between plants possessing tumour-promoting principles and the increased incidence of human tumours in certain geographic regions of the world. Hirayama and Ito (1981) indicated that there is a strong circumstantial link between the high incidence of nasopharyngeal carcinoma (NPC) in the southern regions of China and the distribution of Euphorbiaceae and Thymelaeceae plants. Among these plants, extracts from *Croton tiglium*, *Euphorbia lathylis*, *Aleurites fordii*, *Jatropha curcas*, *Euphorbia antiquorum*, *E. milii*, *E. pekinensis*, *E. kansui* and *Daphne odora* all exert Epstein-Barr virus (EBV)-activating capacity. The active component of the classic tumour promoter of croton oil from *Croton tiglium* L was purified and its chemical structure defined as 12-*O*-tetradecanoyl phorbol-13-acetate (TPA) (Hecker, 1968).

A similar study also indicated a causal link between the regular intake of phorbol esters contained in 'Walensalii tea' prepared from *Croton flaven* L. (Euphorbiaceae) and the high incidence of oesophageal cancer in Curacao Island of the Caribbean (Hecker, 1987).

Burkitt lymphoma (BL), an EBV-associated non-Hodgkin's malignant lymphoma, is endemic in an area of Africa known as the lymphoma belt. In East Africa (Kenya, Uganda and Tanzania), where BL is endemic, over 90 species of Euphorbiaceae plants are reported to be currently used as common folk remedies (Kokwaro, 1979). EBV-activating capacity has been demonstrated in extracts of some of these plants (Ito, 1986).

EBV-activating capacity has also been reported in 11 out of the 12 species of the Euphorbiaceae family commonly grown in the Cameroon, one of the endemic areas of BL (Ohigashi *et al.*, 1985). In Malawi, *E. tirucalli* and other EBV-activating plants were found significantly more often in homes of BL patients than in those of controls (Van den Bosch *et al.*, 1993). Recently, *E tirucalli* has been reported to induce the characteristic 8:14 translocation of endemic BL in EBV-infected lymphoblastic cell lines *in vitro* (Aya *et al.*, 1991).

In Malaysia, herbal medicaments are widely used by the rural Malays. Several parts of the different plants are used. Dried parts of the plants such as roots and barks are usually boiled and the decoction taken internally. Fresh specimens are usually squashed and applied externally as a poultice.

Many of the indigenous plants used are also from the Euphorbiaceae and Thymelaeceae families. NPC, which is associated with EBV, is also common among the Malaysian Malays, although the prevalence is lower than in the Malaysian Chinese (Armstrong *et al.*, 1979; Yadav *et al.*, 1985). It would be interesting to study the incidence of NPC in relation to the use of these plants. However, in this study, we only screen the members of the Euphorbiaceae used by herbalists for their tumour-promoting activity.

It has now been established that latent EBV can be induced and activated from genome-carrying human lymphoblastoid cells *in vitro* by treatment with tumour-promoting phorbol and other diterpene esters (zur Hausen *et al.*, 1979; Ito *et al.*, 1981*a*), and indole alkaloid (Luka *et al.*, 1979; Ito *et al.*, 1981*b*; Yamamoto *et al.*, 1981). In the present study, a short-term *in vitro* assay for detecting tumour-promoting activity using the activation of EBV antigen expression in EBV genome-carrying human lymphoblastoid cells (Raji) has been used as the screening assay (Ito *et al.*, 1981*b*). This assay system is rapid and efficient for detecting EBV-active principles in the environment (Ito *et al.*, 1981*a,c*).

It is also noteworthy that such EBV-activating principles can be considered to possess tumour-promoting capacity (Ito *et al.*, 1981*a*). The assay can thus be used as a screening test in the search for promoter substances in nature.

### Materials and methods

### *Collection of plants*

The Euphorbiaceae species were collected from various places in Malaysia. Some of the plants were collected from the University of Malaya botanical garden, Rimba Ilmu. The rest of the plants were collected during field trips conducted in the Malaysian states of Selangor, Pahang, Kedah and Kelantan. The list of plants collected is shown in Table I.

### *Preparation of crude extract*

Crude ether extracts of dried, powdered plant material were prepared as previously described (Yadav *et al.*, 1989). Ten grams of the powdered material was extracted with ether (10 ml) at room temperature for 48 h. After evaporating the solvent, the resultant residue was weighed and dissolved in dimethyl sulphoxide (DMSO) (Sigma) as a stock solution of 20 $mg\,ml^{-1}$ and kept at $-20°C$ until use.

### *EBV early antigen induction in Raji cells*

The technique used was as previously described (Ito *et al.*, 1981*a*; Yadav *et al.*, 1989). Briefly, rapidly growing human

Correspondence: AW Norhanom
Received 9 August 1994; revised 14 November 1994; accepted 15 November 1994

**Table I** List of botanical names of Euphorbiaceae species screened for Epstein–Barr virus early antigen induction in Raji cells

| | |
|---|---|
| *Acalypha hispida* Burm. f | *Elateriospermum tapos* |
| *A. indica* L | *Euphorbia atoto* |
| *A. siamensis* oliv ex Gage | *E. hirta* L |
| *A. wilkensia* Moorea | *E. tirucalli* L |
| *A. wilkensia* Macafeana | *E. pulcherrima* Willd |
| *Agrostistachys longifolia* | *E. splendes* |
| *A. sessifolia* | *Exocoecaria agallocha* |
| *Antidesma cupidatum* | *Glochidion brunneum* |
| *A. pendulum* | *G. lucidon* |
| *A. salicinum* Ridl | *G. macrostigma* |
| *Aparusa prainiana* King ex Gage | *Homalanthus populneus* |
| *Baccaurea dukis* Muell Arg | *Jatropha gossypyfolia* L |
| *B. lanceolata* (Mig) | *J. podagrica* |
| *B. parviflora* (MA) MA | *Macaranga heynei* |
| *B. scortechinii* | *M. teccurrata* |
| *Breynia coronata* | *M. species* |
| *Cheatocarpus castanocarpus* | *Pedilanthus tithymaloides* (L) Poitt |
| *Chephalomappa malloticarpa* JJ Smith | *Phyllanthus frodosus* Wall |
| *Cicca acadia* | *P. niruri* |
| *C. accidia* Merr | *P. reticulatus* Poirr |
| *Coedium variegatum* (L) Bl | *Portulaca oleraceae* |
| *Croton argyratus* Bl | *Suregada angustifolia* (MA) Air Saw. |
| *C. candalus* | *Sebastiana chamaelea* (L) MA |

lymphoblastoid Raji cells were incubated with plants extracts in the presence of 4 mM sodium butyrate. The cells were incubated for 72 h in a humidified incubator at 37°C with 5% carbon dioxide in air. The positive control consisted of Raji cells treated with optimal concentration (10 ng $ml^{-1}$) of TPA (Sigma) and 4 mM *n*-butyrate (Sigma). The negative control consisted of untreated culture media. After 72 h, the cells were harvested and checked for the presence of EBV early antigen by means of the indirect immunofluorescence assay. Nasopharyngeal carcinoma EBV-positive sera were used to detect the presence of EBV early antigen. The assay was repeated once in order to obtain consistent results.

### Results

Twenty-seven per cent (13 out of 48) of the Euphorbiaceae screened were found to be positive for tumour-promoting activity when tested at concentrations of 10–40 $\mu g\ ml^{-1}$ cell culture medium (see Table II). Crude extract of four plants, namely *Breynia coronata* Hk.f., *Codiaeum variegatum* (L) Bl, *Euphorbia atoto* and *Exocoecaria agallocha*, demonstrated EBV-induced activity even when tested at lower concentrations of between 0.2 and 1.2 $\mu g\ ml^{-1}$ cell culture. Relative to

**Table II** List of Euphorbiaceae species positive for Epstein–Barr virus early antigen (EA) induction in Raji cells

| | | | *Concentration of crude ether extract tested ug $ml^{-1}$* | | | | | |
|---|---|---|---|---|---|---|---|---|
| *Botanical name* | *Medicinal uses* | *Plant part tested* | *0.2* | *0.6* | *1.2* | *10.0* | *20.0* | *30.0* |
| *Breynia coronata* Hk f | Not documented | Leaf | – | – | + | + | + | + |
| *Coaedium variegatum* (L) Bl | (a) Pounded root is rubbed into syphilitic sores[a] (b) Leaves are used to poultice the abdomen of children with urinary problems[a] | Leaf | – | ± | ± | + | + | + |
| *Croton argyratus* Bl | (a) A decoction of the leaves is used as a post-partum protective medicine and a treatment for colic or diarrhoea[b] | Leaf | – | – | – | + | + | + |
| | (b) Leaves prescribed in medicinal bath to treat fever and as an application for ulcers[a] | Leaf | | | | | | |
| *Euphorbia atoto* | Not documented | Leaf | – | – | ± | + | + | + |
| *E. hirta* L | (a) It is made into poultices for sores on legs caused by the marine worm. The latex is dropped into the eyes for conjunctivitis and ulcerated cornea[a] (b) It has a reputation as a remedy for bronchitis and asthma, and is slightly narcotic, hence helping patients to sleep.[a] | Whole plant | – | – | – | + | + | + |
| *E. tirucalli* L | (a) Roots and stems are used as a poultice applied to ulceration of the nose, haemorrhoids, swellings of the nose and painful parts[a] | Stem | – | – | – | + | + | + |
| *E. splendes* | Not documented | Stem | – | – | – | + | + | + |
| *Exocoecaria agallocha* | (a) The latex is used in some places as a caustic in the treatment of | Stem | + | + | + | + | + | + |
| | obstinate ulcer[b] | Leaf | – | – | – | + | + | + |
| | (b) A little of the bark chewed causes vomiting and purgingin and may be resorted to in constipation[b] | Root | – | – | – | – | + | + |
| *Homalanthus populneus* | (a) Oiled leaves are heated and applied to the stomach to treat fever[b] | Twig | – | – | – | + | + | + |
| *Jatropha gossypifolia* | (a) Seeds contain oil which is emetic and purgative[b] | Leaf | – | – | – | + | + | + |
| *J. podagrica* | Not documented | Seed | – | – | – | + | + | + |
| *Pedilanthus titymaloides* (L) Poitt | (a) Applied to skin for leucoderma and for scorpion and centipede bites[b] (b) Leaves and stem also used as a meat tenderiser in cooking | Stem | – | – | – | + | + | + |
| *Suregada augustifolia* (MA) Air Saw | Not documented | Leaf | – | – | – | + | + | + |
| | | Stem | – | – | – | + | + | + |

TPA (20 ng $ml^{-1}$) activated EBV EA in 30% of Raji cells. –, did not activate EBV EA in Raji cells; +, activated EBV EA in >30% of Raji cells; ±, activated EBV EA in <30% of Raji cells. [a]Perry (1980). [b]Burkill (1966).

the other plants. *Exocoecaria agallocha* demonstrated the highest activity since the crude extract remained strongly positive even when tested at $0.2\,\mu g\,ml^{-1}$ cell culture.

### Discussion

Since plant diterpene esters in Euphorbiaceae and Thymelaeceae plants have been found to exert EBV-inducing activity (Ito *et al.*, 1981*b*; Zeng *et al.*, 1983), only ether extracts were tested in the present study. However, the EBV-activating property has also been reported in methanol and water extracts of plants (Zeng *et al.*, 1983). It is to be hoped that other plant extracts will be tested in the future.

To date, no studies have been carried out to relate the use of indigenous plants with cancer in Malaysia. Therefore, the link between tumour promoters and cancers in Malaysia is unknown. From this study, it is disturbing to note the high prevalence of EBV-inducing activity in the species of Euphorbiaceae tested, especially since nasopharyngeal carcinoma, which is associated with EBV, is common among Malaysians (Armstrong *et al.*, 1979; Yadav *et al.*, 1985).

Since many of the EBV inducers are tumour promoters (Ito *et al.*, 1981*b*; Zeng *et al.*, 1983), the hypothesis that these positive plants are the aetiological factors for NPC is valid not only for NPC but also for other malignant tumours. Among the Malays, these plant extracts are either taken internally as a decoction or a concoction or used externally as a poultice. In both ways, there is direct contact between the plant extract and the skin or mucous membrane of the digestive tract. Plant extracts with EBV-activating capacity have been reported to retain their capacity to induce EBV complex in viral genome-carrying human lymphoblastoid cells (Raji) even after heat treatment at 120°C for 2 h or at 100°C for 12 h (Yanase and Ito, 1984). The medicinal plants with tumour-promoting activity could well be an important aetiological factor in the promotion of tumours among Malays who use these plants regularly in folk medication.

Detailed chemical studies on the positive EBV-activating plants identified in this study have not been carried out. However, the active compounds of *J. podagrica* and *J. gossypyfolia* have been reported to be polyunsaturated esters of the tigliane-type diterpenoids 16-hydroxyphorbol and 12-deoxy-16-dydroxyphorbols respectively (Adolf *et al.*, 1984). Highly irritant and promoting principles of *E. tirucalli* have been characterised as 4-deoxyphorbol diesters (Kinghorn, 1979). The active tumour-promoting principle of *E. agallocha* has been shown to be the piscicidal daphnane-type orthoester 'excoecaria toxin', and the structures of the individual daphne-type irritant factors have been elucidated (Wikiyachitra, 1985). Active principles of the other positive plants identified in this study have not been documented.

EBV-activating principles have been documented to be present in *C. variegatum* (Ohigashi *et al.*, 1985), *E. hirta*, *E. splendes* and *P. tithymaloides* (Ito *et al.*, 1984). However, EBV-activating principles in *B. coronata*, *C. argyratus*, *E. atoto*, *H. populneus* and *S. angustifolia* have not been documented in the literature. *E. pulcherrima* did not exhibit EBV-activating principles and a similar observation has been reported previously (Ito *et al.*, 1984).

The multifactorial aetiology of cancer is complex. However, the most important task of basic cancer research always was and still is the development of generally valid criteria for the detection and classification of environmental carcinogens. In Malaysia, good epidemiological studies regarding an association between the use of plants with tumour-promoting activity and the prevalence of cancer (tumours) are clearly needed. More plants should be screened for this activity, and further studies on tumour-promoting activity in Malaysian plants are necessary.

**Acknowledgements**
We would like to thank Ms Afidah Sastro and Mr Muhamad Ilham Adenan for their technical assistance, and Professor Engkit Soepadmo for conducting the field trips. This study was carried out under the auspices of the ASEAN-Australian Biotechnology Project with financial assistance from AIDAB. The authors gratefully acknowledge this support.

### References

ADOLF W, OPFERKUCH HJ AND HECKER W. (1984). Irritant phorbol derivatives from four Jatropha species (Euphorbiaceae). *Phytochemistry*, **23**, 129–132.

ARMSTRONG RW, KANNAN-KUTTY M, DHAMALINGAM SK AND PONNUDURAI JR. (1979). Incidence of nasopharyngeal carcinoma in Malaysia, 1968–1979. *Br. J. Cancer*, **40**, 557–567.

AYA T, KINOSHITA T, IMAI S, KOIZUMI S, MIZUNO F, OSATO T, SATOH C, OIKAWA T, KUZUMAKI N, OHIGASHI H AND KOSHIMIZU K. (1991). Chromosome translocation and c-myc activation by Epstein–Barr virus and *Euphorbia tirucalli* in B lymphocytes. *Lancet*, **i**, **337**, 1190.

BURKHILL IH. (1966). *A Dictionary of the Economic Products of the Malay Peninsula*. **Vols 1 and 2**. Ministry of Agriculture Cooperative: Kuala Lumpur, Malaya.

HECKER E. (1968). Cocarcinogenic principles from the seed oil *Croton tiglium* L. and from other Euphorbiaceae. *Cancer Res.*, **28**, 2338–2349.

HECKER E. (1987). Tumour promoters of cancer irritant diterpene esters type as risk factors of cancer in man. *Bot. J. Linn. Soc.*, **94**, 197–219.

HIRAYAMA T AND ITO Y. (1981). A new view of the etiology of nasopharyngeal carcinoma. *Prev. Med.*, **10**, 614–622.

ITO Y. (1986). Vegetable activators of the viral genome and the causation of Burkitt's lymphoma and Nasopharyngeal Carcinoma. In *The Epstein–Barr Virus: Recent Advances*. Epstein MA, and Achong BG (eds) pp. 207–236. William Heinemann Medical Books: London.

ITO Y, YANASE S, FUJITA J, HIRAYAMA T, TAKASHIMA M AND IMANAKA H. (1981*a*). A short-term *in vitro* assay for promoter substances using human lymphoblastoid cells latently infected with Epstein–Barr virus. *Cancer Lett.*, **13**, 29–37.

ITO Y, KAWANISHI M, HIRAYAMA T AND TAKABAYASHI S. (1981*b*). Combined effects of the extracts from *Croton tilium*, *Euphorbia lathyris* or *Euphorbia tirucalli* and *n*-butyrate on Epstein–Barr virus expression in human lymphoblastoid P3HR-1 and Raji cells. *Cancer Lett.*, **12**, 175–180.

ITO Y, KISHISHITA M, MORIGAKI T, YANASE S AND HIRAYAMA T. (1981*c*). Induction and intervention of Epstein–Barr virus expression in human lymphoblastoid cell lines: a simulation model for study of cause and prevention of nasopharyngeal carcinoma and Burkitt's lymphoma. In *Nasopharyngeal Carcinoma*, Cancer Campaign, Vol. 5, Grundmann E, Krueger GRF and Ablashi DV (eds) pp. 255–262. Gustav Fisher: Stuttgart.

ITO Y, TOKUDA H, OHIGASHI H AND KASHIMIZU K. (1984). Distribution and characterisation of environmental promoter substances as assayed by synergistic Epstein–Barr virus-activating system. In *Cellular Interactions by Environmental Tumour Promoter*, Fujiki H, Hecker E, Moore RE, Sugimura T and Weinstein IB (eds) pp. 125-137. Japan Scientific Press: Tokyo and VNU Scientific Press: Utrecht.

KINGHORN AD. (1979). Characterisation of an irritant 4-deoxyphorbol diester from *Euphorbia tirucalli*. *J. Natl. Prod.*, **42**, 112–115.

KOKWARO JO. (1979). Euphorbiaceae. In *Medicinal Plants of East Africa*, Kokwaro JO (ed.) pp. 85–100. East Africa Literature Bureau: Nairobi.

LUKA J, KALLIN B AND KLEIN G. (1979). Induction of the Epstein–Barr virus (EBV) cycle in latently infected cells by n-butyrate. *Virology*, **94**, 228–231.

OHIGASHI H, KOSHIMIZU K, TOKUDA H, HIRAMATSU S, JATO J AND ITO Y. (1985). Epstein–Barr virus – inducing activity of Euphorbiaceae plants commonly grown in Cameroon. *Cancer Lett.*, **28**, 135–141.

PERRY LM. (1980). Medicinal plants of East and South East Asia. MIT Press: Cambridge, MA, USA.

VAN DEN BOSCH C, GRIFFIN BE, KAZEMBE P, DZIWENI C AND KADZAMIRA L. (1993). Are plant factors a missing link in the evolution of endemic Burkitt lymphoma? *Br. J. Cancer*, **68**, 1232–1235.

WIRIYACHITRA P, HAJIWANGOH H, BOONTON P, ADOLF W, OPFERKUCH HJ AND HECKER E. (1985). Investigations of medicinal plants of Euphorbiaceae and Thymelaeceae occurring and used in Thailand. II. Cryptic irritants of the diterpene ester type from three Excoecaria species. *Planta Med.*, **5**, 357–472.

YADAV M, TAN MK AND SINGH P. (1985). Nasopharyngeal carcinoma in Malaysia: distribution by race for period 1981–1983. *J. Malays. Soc. Health*, **5**, 67–71.

YADAV M, ILHAM M AND NORHANOM AW. (1989). Epstein–Barr virus early antigen induction in Raji cells by plants used in Malaysian traditional medicine. *Asean J. Clin. Sci.*, **9**, 71–77.

YAMAMOTO H, KATSUKI T, HINUMA Y, HOSHINO H, MIWA M, FUJIKA H AND SUGIMURA T. (1981). Induction of Epstein–Barr virus by a new tumour promoter, teleocidin, compared to induction by TPA. *Int. J. Cancer*, **28**, 125–129.

YANASE S AND ITO Y. (1984). Heat durability of Epstein–Barr virus activating substances of plant origin: 13-O-tetradecanoylphorbol-13-acetate, 12-O-hexadecanoyl-16-hydrophorbol-13-acetate, Croton oil, tung oil and *Croton megalocarpus* extract. *Cancer Lett.*, **22**, 183–186.

ZENG Y, ZHONG JM, MO YK AND MIAO XC. (1983). Epstein–Barr virus early antigen induction in Raji cells by Chinese medicinal herbs. *Intervirology*, **19**, 201–204.

ZUR HAUSEN H, BORNKAMM GW, SCHMIDT R AND HECKER E. (1979). Tumour initiators and promoters in the induction of Epstein–Barr virus. *Proc. Natl Acad. Sci. USA*, **76**, 782–785.